# Supplementary material for: Role of bulge epidermal stem cells and TSLP signaling in psoriasis
Source: EMBO Mol Med. 2019 Sep 26;11(11):e10697. doi: 10.15252/emmm.201910697 (PMC6835205; doi:10.15252/emmm.201910697)
Supplement: Supplementary file 1 — Appendix [file EMMM-11-e10697-s001.pdf]

Role of bulge epidermal stem cells and TSLP signaling in psoriasis

Nuria Gago-Lopez et al.

APPENDIX FIGURES AND LEGENDS

Table of contents:

- **Appendix Figure S1.** Characterization of DKO*-mT/mG mice during psoriasis-like progression.
- **Appendix Figure S2.** Dynamic pattern of GFP/Tomato epidermal cells in tail and back skin of DKO*-mT/mG mice during psoriasis-like progression.
- **Appendix Figure S3.** FACS analyses of different epidermal stem cell populations in ear skin of Co-mT/mG and DKO*-mT/mG mice during psoriasis-like progression.
- **Appendix Figure S4.** CD34 expression in bulge HF-SCs of DKO*-mT/mG mice by immunofluorescence and FACS strategy for sorting.
- **Appendix Figure S5.** Mutant^{GFP} keratinocytes induce hyper-proliferation in control^{Tom} keratinocytes.
- **Appendix Figure S6.** Characterization of immune cell infiltration and TSLP signaling in DKO* and DKO*^{15} mice treated with anti-TSLP.
- **Appendix Table S1. Primers for qPCR.**
- **Appendix Table S2. List of P-values.**

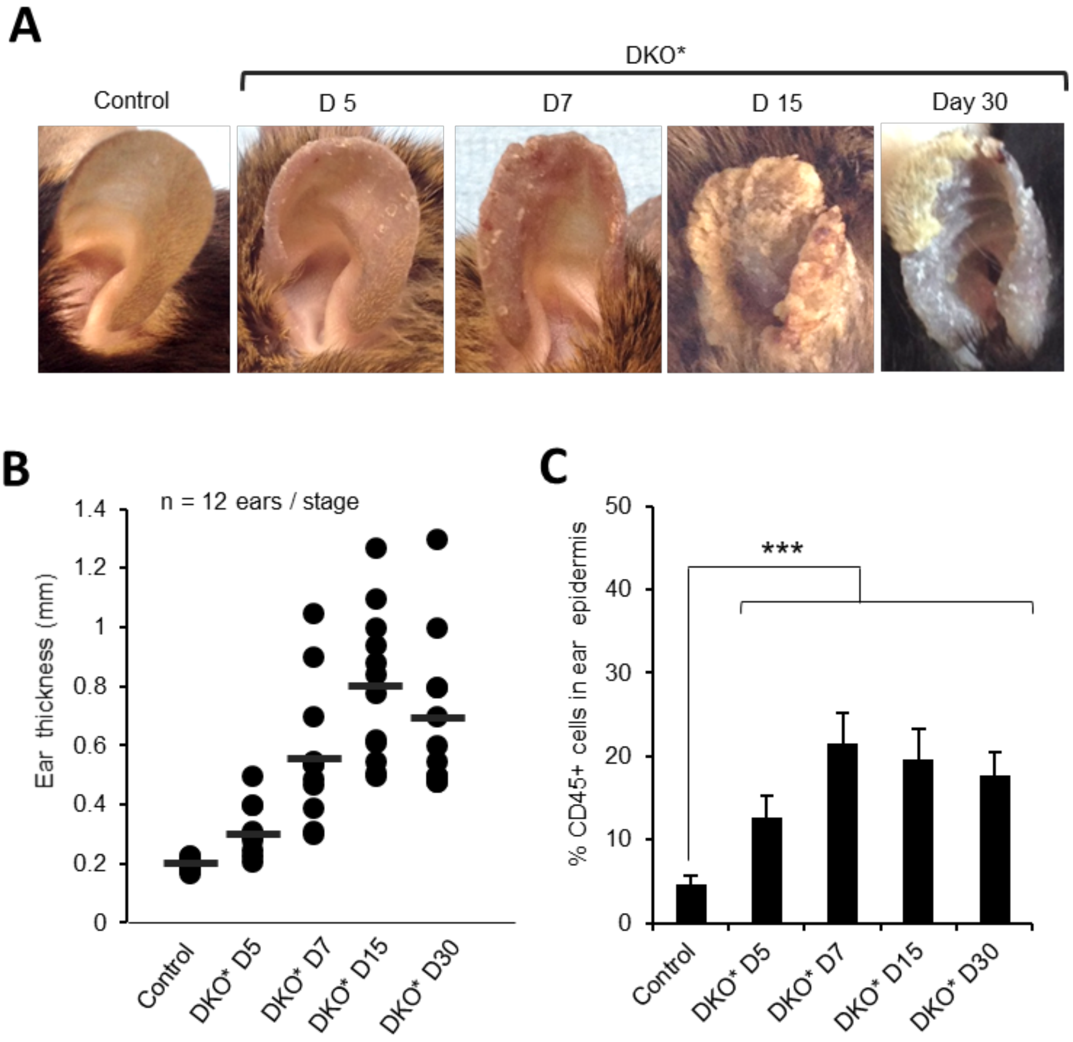

Appendix Fig S1. Characterization of DKO*-mT/mG mice during psoriasis-like progression.

A. Images of ear skin at different time points during psoriasis-like development. Same control ear image was included in Fig. 3B.

B. Ear thickness measurement at different time points during psoriasis-like development.

C. FACS quantification of $CD45^+$ cells from ear skin of control and DKO* mice at different time points during psoriasis-like development. n = 5-10 per time point. Statistical significance ***p<0.0001 (t-student two tailed-test relative to control group). See Supplemental Appendix Table 2 for exact p-Value.

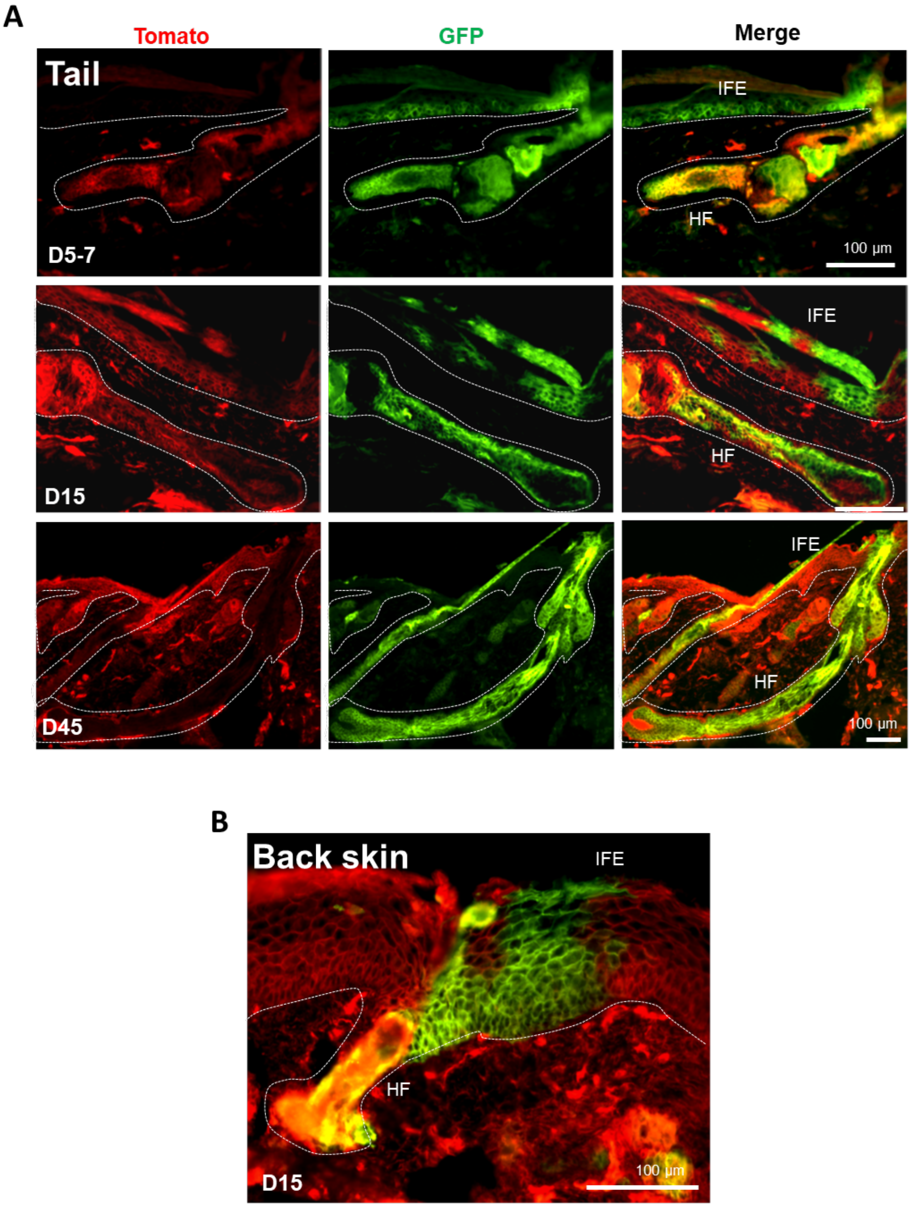

Appendix Fig S2. Dynamic pattern of GFP/Tomato epidermal cells in tail and back skin of DKO*-mT/mG mice during psoriasis-like progression.

A. Fluorescence imaging of DKO*-mT/mG tail skin at different time points during psoriasis-like progression. White dotted line separates epidermis and dermis.

B. Fluorescence imaging of DKO*-mT/mG back skin at day 15 after tamoxifen treatment. White dotted line separates epidermis and dermis.

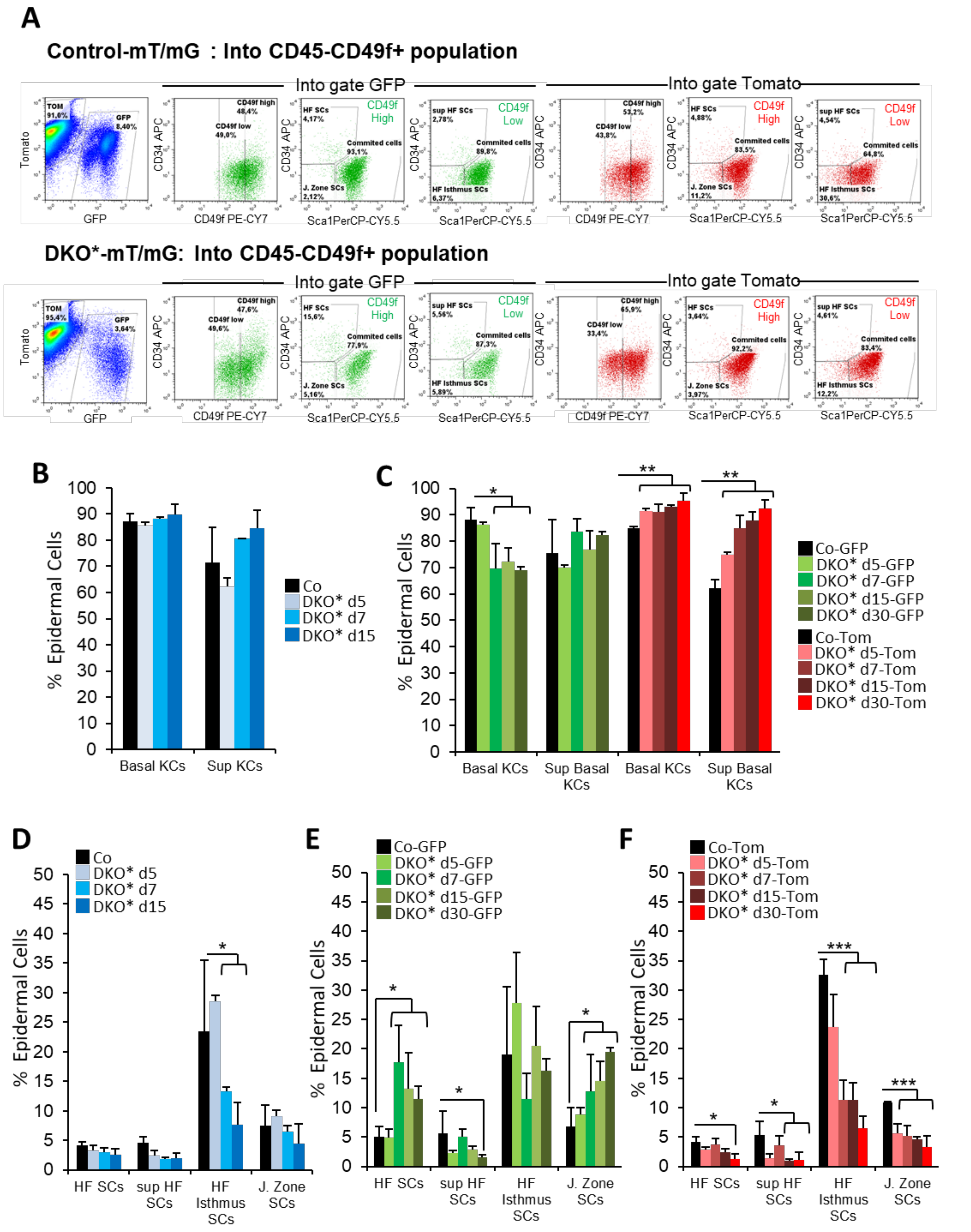

Appendix Fig S3. FACS analyses of different epidermal stem cell populations in ear skin of Co-mT/mG and DKO*-mT/mG mice during psoriasis-like progression.

A. Strategy for FACS analysis of different epidermal stem cell populations in Co-mT/mG and DKO*-mT/mG mice.

B. FACS quantification of basal and suprabasal keratinocytes from IFE at different time points during psoriasis-like development. n=3 per time point. Statistical significance *p<0.05 (t-student two tailed-test relative to control groups). See Supplemental Appendix Table 2 for exact p-Value.

C. FACS quantification of basal and suprabasal keratinocytes from IFE into GFP^+ and $Tomato^+$ gating at different time points during psoriasis-like development. Reduction in $mutant^{GFP}$ IFE basal keratinocytes and enrichment of non-$mutant^{Tom}$ IFE populations. Statistical significance *p<0.05 (t-student two tailed-test relative to control groups). See Supplemental Appendix Table 2 for exact p-Value.

D. FACS quantification of HF-SC subpopulations at different time points during psoriasis-like development. n=3 per time point. Statistical significance *p<0.05 (t-student two tailed-test relative to control groups). See Supplemental Appendix Table 2 for exact p-Value.

E. FACS quantification of HF-SC subpopulations into GFP^+ gating at different time points during psoriasis-like development. Enrichment of bulge HF-SCs and junctional zone SCs. Statistical significance *p<0.05 (t-student two tailed-test relative to control groups). See Supplemental Appendix Table 2 for exact p-Value.

F. FACS quantification of HF-SC subpopulations into $Tomato^+$ gating at different time points during psoriasis-like development. Reduction in all HF-SC subpopulations along psoriasis-like progression. Statistical significance *p<0.05 (t-student two tailed-test relative to control groups). See Supplemental Appendix Table 2 for exact p-Value.

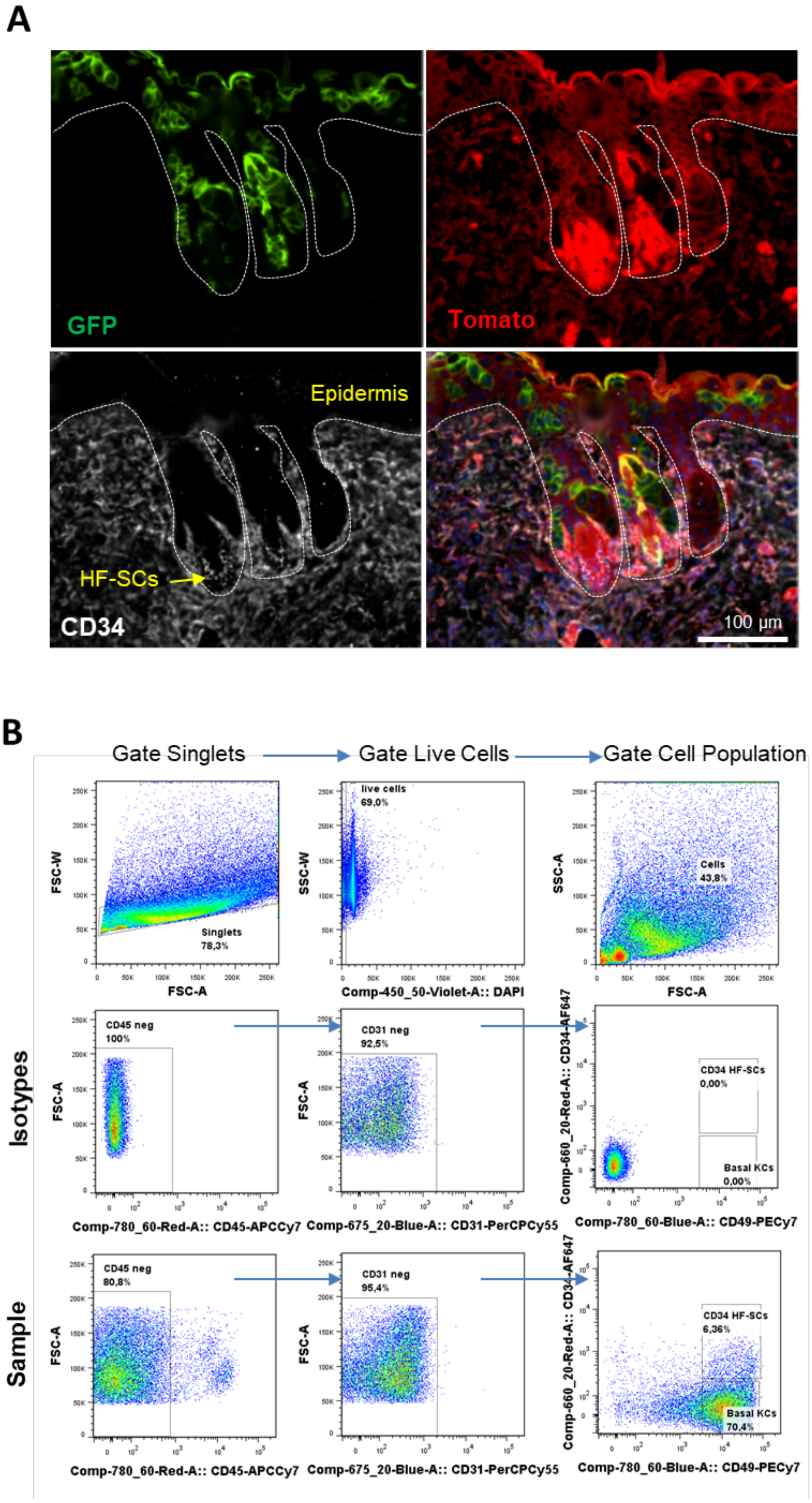

Appendix Fig S4. CD34 expression in bulge HF-SCs of DKO*-mT/mG mice by immunofluorescence and FACS strategy for sorting.

A. Immunofluorescence image of CD34 staining in ear skin of psoriatic DKO*-mT/mG mice. White dotted line separates epidermis and dermis.

B. Strategy for FACS sorting of CD34 bulge HF-SCs and CD49f basal KCs. Sorting procedures were done excluding dead cells and doublets. Cell population positive for CD45 and CD31 were discarded and cells positive for $CD34^{+}/CD49f^{high}$ and $CD34^{-}/CD49f^{high}$ were sorted for further experiments.

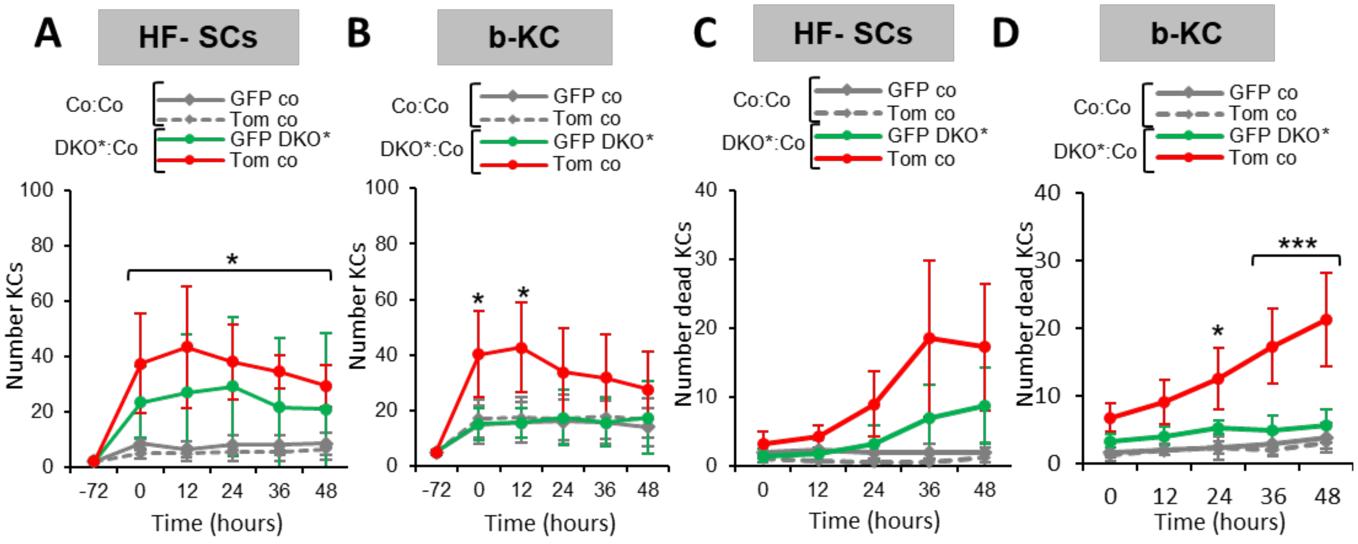

Appendix Fig S5. Mutant^{GFP} keratinocytes induce hyper-proliferation in control^{Tom} keratinocytes.

A, B. Average of total number of live and dead keratinocytes per field from co-cultures of mutant or control GFP^{+} HF-SCs with control Tomato^{+} HF-SCs at different time points of time lapse capture during 48 hours. n = 10-12 fields analyzed in total from two independent experiments. Statistical significance *$p<0.05$ (Two-way ANOVA and Bonferroni post test).

C, D. Average of total number of live or dead keratinocytes per field from co-cultures of mutant or control GFP^{+} basal keratinocytes with control Tomato^{+} basal keratinocytes at different time points of time lapse capture during 48 hours. n = 4 fields from two independent experiments. Statistical significance *$p<0.05$, ***$p<0.001$ (Two-way ANOVA and Bonferroni post test).

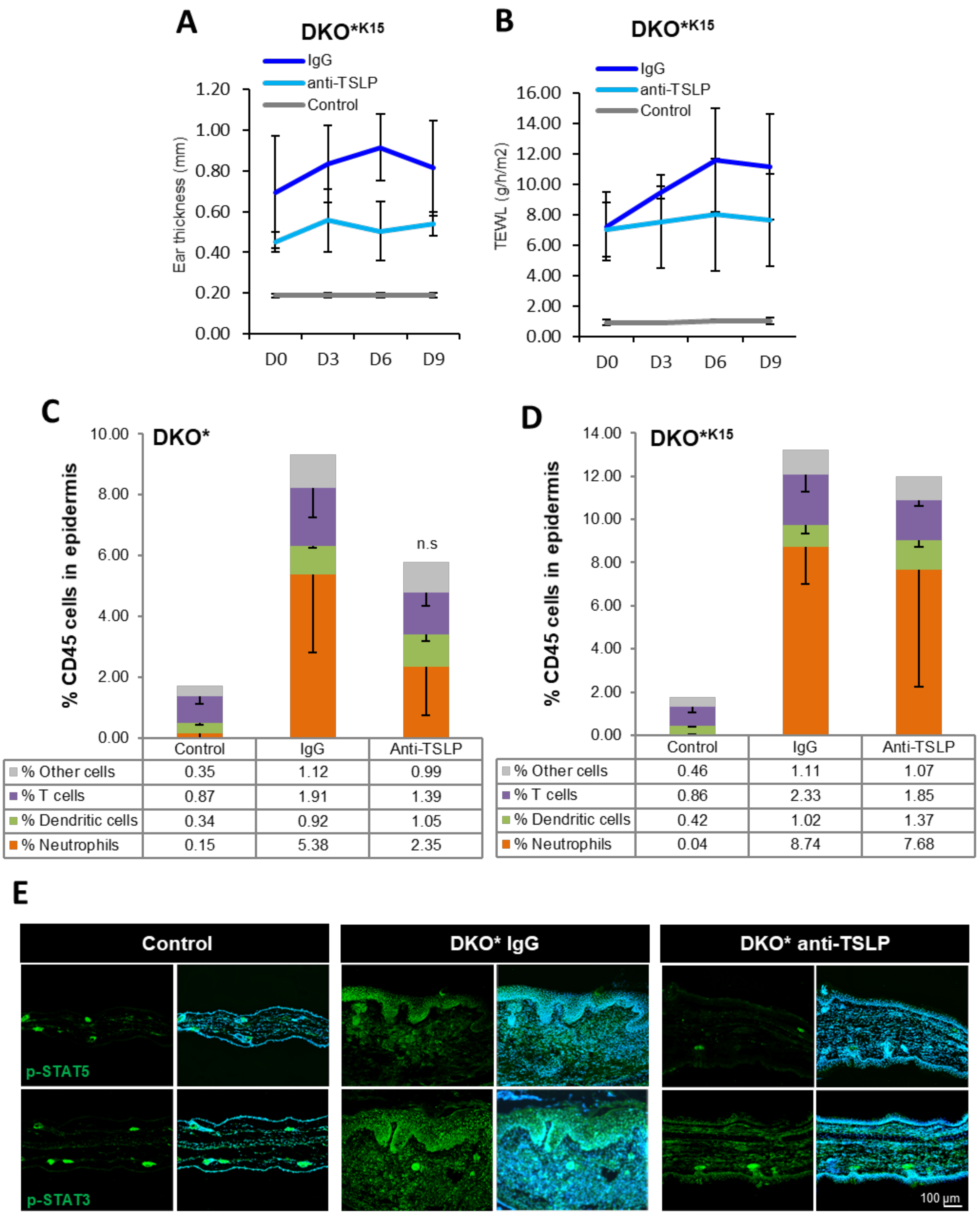

Appendix Fig S6. Characterization of immune cell infiltration and TSLP signaling in DKO* and DKO*^{15} mice treated with anti-TSLP.

A, B. Longitudinal analysis of ear thickness (I) and Transepidermal water loss (TEWL) (J) in DKO*[15] mice at different time points during psoriasis-like disease progression (Two-way ANOVA and Bonferroni post test, n=3, n.s = non statistical significance).

C, D. FACS quantification of immune cell subtypes (T cells, dendritic cells and neutrophils) on ear skin of DKO* and DKO*[15] mice after anti-TSLP or IgG treatment by FACS analysis. After anti-TSLP treatment, neutrophils are reduced in DKO* mice. DKO* n=4, DKO*[15] n=3. n.s = non statistical significance (t-student two tailed-test relative to control groups).

E. Representative immune-fluorescence images of ear sections from DKO* mice treated with IgG (left panel) or anti-TSLP (right panel) stained for p-STAT5 and p-STAT3. p-STAT5 expression was inhibited in mice treated with anti-TSLP, whereas p-STAT3 expression was maintained in both groups, IgG and anti-TSLP (n =3).

Table S1. Primers for qPCR

Name	Sequence
Tslp-F	GAGGACTGTGAGAGCAAGCCAG
Tslp-R	GGCAGTGGTCATTGAGGGCTT
Tslpr-F	CGGGAGAGCAATGACGATG
Tslpr-R	CCGAACCCGGAAGTCATAGC
IL-7rα-F	TGTGAGTTTCAATCCCGAAAGT
IL-7rα-R	CTGGCTGTGCAGGAAGATCA
Vegfα-F	CAGTCCGAGCCGGAGAGGGAGC
Vegfα-R	CGGACGGCAGTAGCTTCGCTGG
Foxc1a-F	CACTCGGTGCGGGAAATGT
Foxc1a-R	GTGCGGTACAGAGACTGACTG
Nfatc1-F	GGCGGGAAGAAGATGGTGCTGTC
Nfatc1-R	TGGTTGCGGAAAGGTGGTATCTCA
Ptgs2-F	AAGCCGAGCACCTTTGGAG
Ptgs2-R	ATTGATGGTGGCTGTTTTGGTAG
Tnfα-F	CCCCAAAGGGATGAGAAGTT
Tnfα-R	CACTTGGTGGTTTGCTACGA
Il-23a-F	AGCGGGACATATGAATCTACTAAGAGA
Il-23a-R	GTCCTAGTAGGGAGGTGTGAAGTTG
Il-1α-F	GGCTCACTTCATGAGACTTGC
Il-1α-R	AGGTGTAAGGTGCTGATCTGG
Il-6-F	CCGGAGAGGAGACTTCACAG
Il-6-R	CAGAATTGCCATTGCACAAC
Il-1β-F	GGGCTGGACTGTTTCTAATGCCTT
Il-1β-R	CCATCAGAGGCAAGGAGGAAAACA
p65-F	CCAAAAAGACGTGCCTCCTG
p65-R	CCCCCAGCCCATAGGGAAC
G-CSF-F	CTGCCACCATCCCTGCCTCT
G-CSF-R	CCATCTGCTGCCAGATGGTGGT
IFN-γ-F	GAAAATCCTGCAGAGCCAGATT
IFN-γ-R	TGATGGCCTGATTGTCTTTCAA
S100a9-F	GTTTGTGTCCAGGTCCTCC
S100a9-R	CACCTTCTCAGATGGAGCG

Table S2. List of p-Values

Figure		sample number	exact p-Value
1	I	n = 2-6	Co vs Lesional: *=0.021; Non Lesional vs Lesional: **=0.0044
1	J	n = 2-6	Co vs Lesional: **=0.01; Non Lesional vs Lesional: ***=0.0007
2	D	n = 6	IFE Thickness. Co vs DKO* D5: n.s =0.055; Co vs DKO* D7: ** =0.0015; Co vs DKO* D15: *** =0.0001; Co vs DKO* D30: *** =0.0002
2	E	n = 3	cCas3. DKO*GFP vs DKO*Tom D5: n.s =0.13; DKO*GFP vs DKO*Tom D7: * =0.044; DKO*GFP vs DKO*Tom D15: n.s =0.79
2	E	n = 3	Ki67. DKO*GFP vs DKO*Tom D5: n.s =0.87; DKO*GFP vs DKO*Tom D7: *** =0.0007; DKO*GFP vs DKO*Tom D15: * =0.05
3	D	n = 5-12	Co vs DKO* D5: * =0.014; Co vs DKO* D7: ** =0.0065; Co vs DKO* D15: *** =0.0005; Co vs DKO* D30: ** =0.0027; Co vs DKO15* D5: ** =0.0032; Co vs DKO15* D7: *** =0.0001; Co vs DKO15* D15: ** =0.0014; Co vs DKO15* D30: *** =0.0001
3	E	n = 4-6	Co vs DKO* D5, D15, D30: ***< 0.00001; Co vs DKO15* D5, D15, D30: ***< 0.00001
3	F	n = 6-10	Co vs DKO* : ***=0.0003; Co vs DKO15* : ***< 0.0001
3	G	n = 4-5	Co vs DKO* : *=0.039; Co vs DKO15* : *= 0.035
3	H	n = 4-5	Co vs DKO* : **=0.0096; Co vs DKO15* : **= 0.0038
3	I	n = 3-6	GFP. Co vs DKO* D5: n.s =0.73; Co vs DKO* D15: ** =0.0028; Co vs DKO* D30: ** =0.0027; Co vs DKO15* D5: ** =0.0026; Co vs DKO15* D15: ** =0.0036; Co vs DKO15* D30: ** =0.003
3	K	n = 3-5	cCas3. DKO15*GFP vs DKO15*Tom D5: n.s =0.2; DKO15*GFP vs DKO15*Tom D7: n.s =0.119; DKO15*GFP vs DKO15*Tom D15: ** =0.0027
3	K	n = 3-5	Ki67. DKO15*GFP vs DKO15*Tom D5: n.s =0.06; DKO15*GFP vs DKO15*Tom D7: * =0.012; DKO15*GFP vs DKO15*Tom D15: *** =0.0001
5	D	n = 3	Co CD34 vs CD34GFP D7: **=0.0047; Co CD34 vs CD34GFP D30: *=0.05; Co CD34 vs CD34Tom D7: **=0.0031; Co CD34 vs CD34Tom D30: **=0.0025; Co CD49f vs CD49fGFP D7: *=0.02; Co CD49f vs CD49fGFP D30: *=0.05
5	E	n = 2	Co CD34 vs CD34GFP D7: *=0.017; Co CD34 vs CD34GFP D30: *=0.05; Co CD34 vs CD34Tom D7: *=0.016; Co CD34 vs CD34Tom D30: *=0.011; Co CD49f vs CD49fGFP D7: *=0.011; Co CD49f vs CD49fGFP D30: *=0.012
5	F	n = 4	Co vs DKO*: **=0.0018; Co vs DKO15*: *=0.04
6	B	n = 3	Ad-Empty vs Ad-Cre: ***<0.0001
6	D	n = 3	IgG vs AbTSLP Ad-Empty: *=0.02; IgG vs AbTSLP Ad-Cre: *=0.05
6	E	n = 3	IgG vs AbTSLP Ad-Cre: *=0.05
6	F	n = 3	IgG vs AbTSLP Ad-Empty: **=0.007; IgG vs AbTSLP Ad-Cre: *=0.011
6	K	n = 3	IgG vs AbTSLP DKO*: *=0.03; IgG vs AbTSLP DKO15*: *=0.015
7	B	n = 3	Control or non Lesional vs Lesional: ***=0.001
7	C	n = 3	Control or non Lesional vs Lesional: *=0.04
EV3	B	n = 3	Co vs DKO* D15: ***=0.0001
EV3	C	n = 3	Co vs DKO* D15: ***=0.0001
EV3	D	n = 3	Co CD34 vs CD34GFP D7: **=0.007
EV4	F	n = 3-4	Co CD34 vs CD34GFP: *=0.015; Co CD34 vs CD34Tom: n.s=0.089
EV4	G	n = 3-4	Co CD34 vs CD34GFP: *=0.021
EV4	H	n = 3	Co CD34 vs CD34GFP: *=0.017; Co CD34 vs CD34Tom: *=0.016
EV4	I	n = 3	Co CD34 vs CD34GFP: *=0.035
EV4	J	n = 3	Co CD34 vs CD34Tom: *=0.024; Co CD49f vs CD49fTom: n.s=0.18
EV4	K	n = 3	Co CD49f vs CD49fTom: **=0.0077
EV4	L	n = 3	All cell subpopulations in relation with the control: **<0.0011
EV5	E	n = 2	Ad-cre IgG GFP vs Ad-cre AbTSLP GFP: *=0.047
EV5	F	n = 2	Ad-cre IgG GFP vs Ad-cre AbTSLP GFP: *=0.05
EV5	G	n = 2	Ad-cre IgG GFP vs Ad-cre AbTSLP GFP: n.s=0.2; Ad-cre IgG Tom vs Ad-cre AbTSLPTom: *=0.05
EV5	H	n = 2	Ad-cre IgG Tom vs Ad-cre AbTSLPTom: n.s=0.08
EV5	I	n = 2	Ad-cre IgG GFP vs Ad-cre AbTSLP GFP: *=0.05
EV5	J	n = 2	Ad-cre IgG GFP vs Ad-cre AbTSLP GFP: *=0.05
EV5	L	n = 2	Ad-cre IgG GFP vs Ad-cre AbTSLP GFP: *=0.029
Appendix S1	C	n = 5-10	Co vs DKO* D5, D15, D30: ***< 0.00001
Appendix S3	C	n = 3	b-KCs Co vs b-KCs GFP D7-30: *=0.042; b-KCs Co vs b-KCs Tom D7-30: **=0.003; Supb-KCs Co vs Supb-KCs Tom D7-30: **=0.0012
Appendix S3	D	n = 3	HF Isthmus SCs Co vs HF Isthmus SCs D7-15: *=0.05
Appendix S3	E	n = 3	HF-SCs Co vs HF-SCs GFP D7-30: *=0.03; Sup HF-SCs Co vs Sup HF-SCs GFP D30: *=0.05; J. Zone SCs Co vs J. Zone SCs GFP D30: *=0.012
Appendix S3	F	n = 3	HF-SCs Co vs HF-SCs Tom D30: *=0.04; Sup HF-SCs Co vs Sup HF-SCs Tom D15-30: *=0.05; HF Isthmus SCs Co vs HF Isthmus SCs Tom D7-30: ***=0.0006; J. Zone SCs Co vs J. Zone SCs Tom D30: ***=0.0004
