# Supplementary material for: Role of bulge epidermal stem cells and TSLP signaling in psoriasis
Source: EMBO Mol Med. 2019 Sep 26;11(11):e10697. doi: 10.15252/emmm.201910697 (PMC6835205; doi:10.15252/emmm.201910697)
Supplement: Supplementary file 2 — Expanded View Figures PDF [file EMMM-11-e10697-s002.pdf]

Expanded View Figures

Figure EV1. Characterization of c-JUN/JUNB in distinct hair follicle stem cell populations from scalp psoriasis patients (related to Fig 1).

A Confocal images of the bulge region of human psoriatic hair follicles from non-lesional and lesional regions of the scalp. CD200 (green), c-JUN (red), DAPI (blue).

B, C Representative composite immunofluorescence images of whole hair follicle units by confocal from non-lesional and lesional scalp psoriasis patients (B) and magnification images of specific regions from the HFs (C). c-JUN (green), JUNB (gray), GATA-6 (red), and DAPI (blue). Yellow arrows represent the outer root sheath basal layer in the bulge (R1, R3) and proximal bulb (Pb, R2, R4). Yellow asterisks represent overexpression of GATA-6 in suprabasal layers of the outer root sheath in lesional bulge region.

 © 2019 The Authors

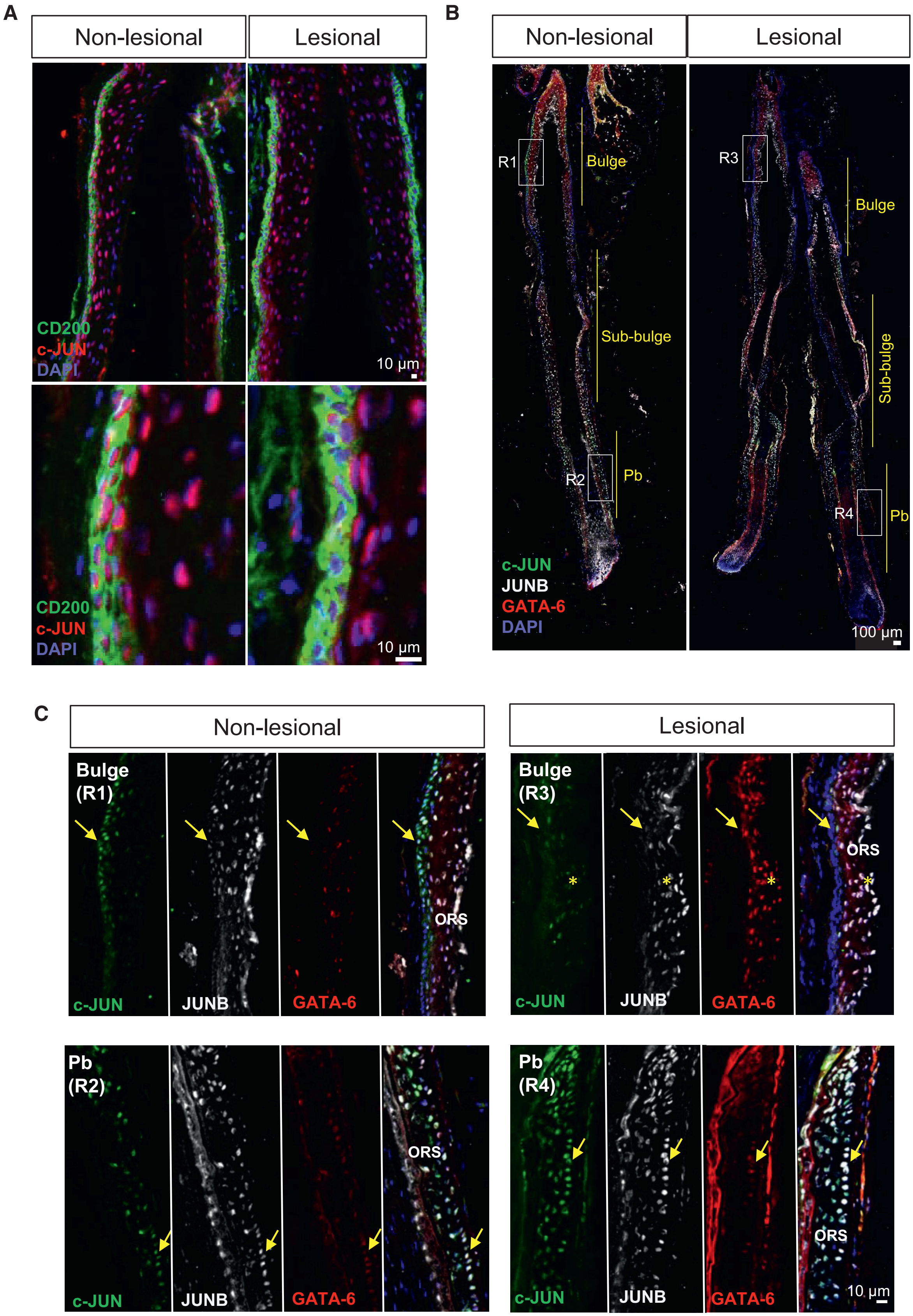
A
Non-lesional
Lesional
CD200
c-JUN
DAPI
10 µm
CD200
c-JUN
DAPI
10 µm
B
Non-lesional
Lesional
R1
Bulge
R2
Sub-bulge
Pb
R3
Bulge
Sub-bulge
R4
Pb
c-JUN
JUNB
GATA-6
DAPI
100 µm
C
Non-lesional
Lesional
Bulge
(R1)
ORS
c-JUN
JUNB
GATA-6
Bulge
(R3)
ORS
c-JUN
JUNB
GATA-6
Pb
(R2)
ORS
c-JUN
JUNB
GATA-6
Pb
(R4)
ORS
c-JUN
JUNB
GATA-6
10 µm

Figure EV1.

Figure EV2. Validation of the lineage tracing system in Co-mT/mG and DKO*-mT/mG mice (related to Fig 2).

A Whole mount of ear skin from co-mT/mG mice before (VEHICLE) and after tamoxifen at day 15. White arrows represent isolated spots of GFP in vehicle skin.

B Immunofluorescence images of Co-mT/mG ear skin sections and DKO*-mT/mG at day 15 after tamoxifen treatment, co-stained with K5 (white). Yellow dotted line separates epidermis and dermis.

C Immunofluorescence images of c-Jun and JunB staining in DKO*-mT/mG at day 15 after tamoxifen treatment showing GFP^+ keratinocytes are negative for c-Jun and JunB expression. GFP (green), Tomato (red), JunB and c-Jun (white), DAPI (blue). Yellow dotted line separates epidermis and dermis.

D Representative images of whole mounts of ear skin from DKO*-mT/mG at day 0 after tamoxifen treatment in two views, from the epidermis and from the dermis, to distinguish the expression of GFP^+ epidermal cell into the IFE and HFs.

© 2019 The Authors

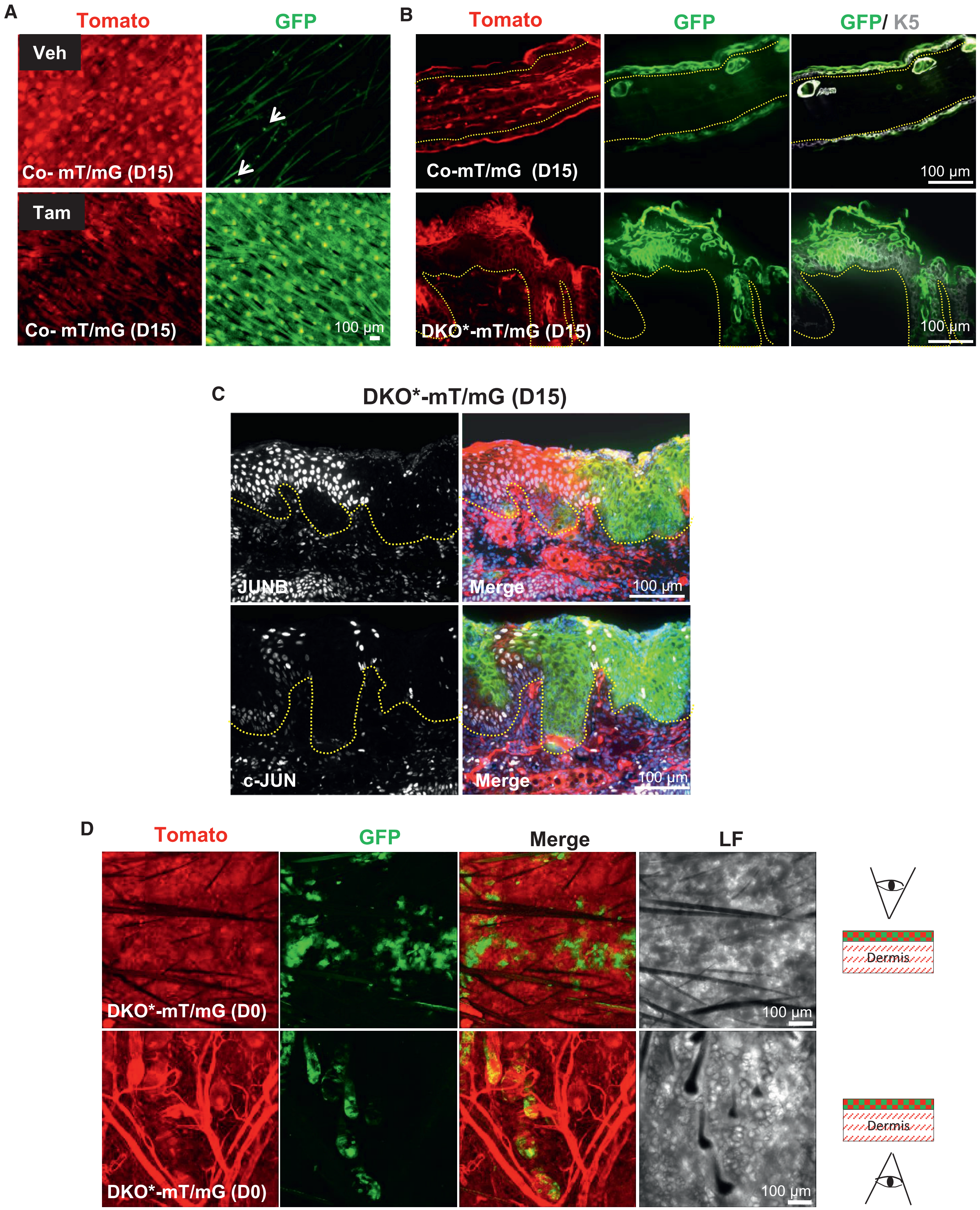
A
Tomato
GFP
Veh
Co- mT/mG (D15)
Tam
Co- mT/mG (D15)
100 µm
B
Tomato
GFP
GFP/ K5
Co-mT/mG (D15)
100 µm
DKO*-mT/mG (D15)
100 µm
C
DKO*-mT/mG (D15)
JUNB
Merge
100 µm
c-JUN
Merge
100 µm
D
Tomato
GFP
Merge
LF
DKO*-mT/mG (D0)
100 µm
Dermis
DKO*-mT/mG (D0)
100 µm
Dermis

Figure EV2.

Figure EV3. Mutant^{GFP} bulge HF-SCs are active and initiate psoriasis-like development (related to Fig 3).

A Immunofluorescence images of the ears of control (Co) and DKO* mice showing co-expression of mutant^{GFP} with CD34 (red arrows) in HF-SCs. n = 3. Scale bar = 50 μm.

B, C Gene expression of quiescence transcription factors, Foxc1a and Nfatc1 show that HF-SCs exit the quiescent stage during psoriasis-like development in DKO* mice. n = 3 mice per group. Data represent mean ± SD. Statistical significance ***$P < 0.001$ (Student's two-tailed t-test relative to control group). See Appendix Table S2 for exact P-values.

D Colony formation *in vitro* of bulge hair follicle stem cells (HF-SCs, CD34$^+$ CD49f^{high}) vs. basal keratinocytes (b-KCs, CD49f^{high}) from control and DKO*-mT/mG ears shows that mutant^{GFP} HF-SCs have significant increased colony formation when compared to non-mutant^{Tom} HF-SCs or control HF-SCs. n = 3 mice per group. Data represent mean ± SD. Statistical significance **$P < 0.01$ (Student's two-tailed t-test relative to control groups). See Appendix Table S2 for exact P-values.

E Representative images of whole mount of ear skin from DKO*^{K15}-mT/mG at day 0 after mifepristone treatment in two views from the epidermis and from the dermis, to distinguish the expression of GFP$^+$ epidermal cell in IFE and HFs.

F Immunofluorescence images of c-Jun and JunB staining in DKO*^{K15}-mT/mG at days 5–7 after tamoxifen treatment show GFP$^+$ keratinocytes are negative for c-Jun and JunB expression.

G Representation of both epidermal lineage-tracking system, DKO*-mT/mG and DKO*^{K15}-mT/mG, for investigating the origin and contribution of epidermal stem cells in the development of psoriasis-like.

 © 2019 The Authors

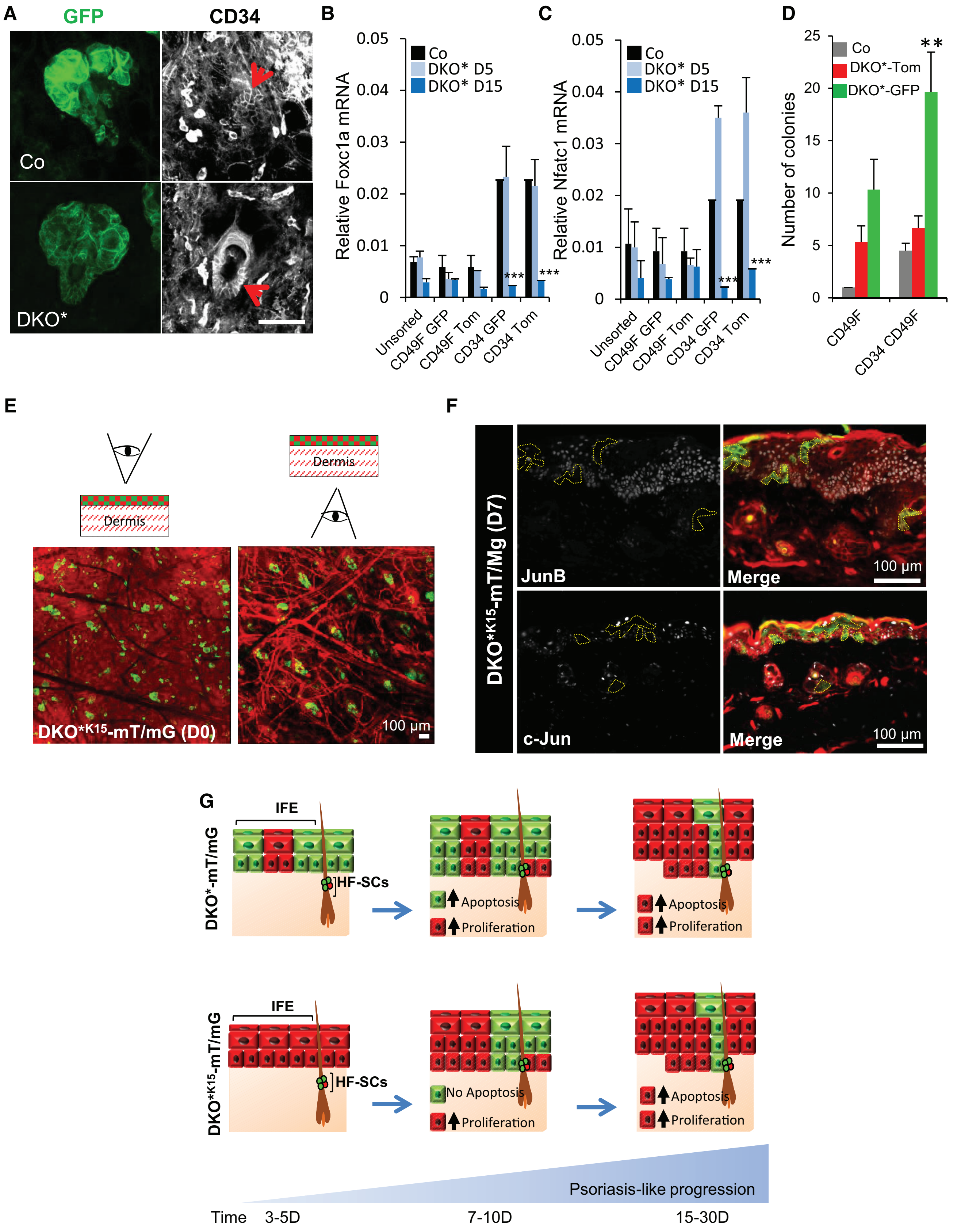
A
GFP
CD34
Co
DKO*
B
Relative Foxc1a mRNA
Co
DKO* D5
DKO* D15
Unsorted
CD49F GFP
CD49F Tom
CD34 GFP
CD34 Tom
C
Relative Nfatc1 mRNA
D
Number of colonies
Co
DKO*-Tom
DKO*-GFP
CD49F
CD34 CD49F
E
Dermis
DKO*K15-mT/mG (D0)
100 µm
F
DKO*K15-mT/Mg (D7)
JunB
Merge
c-Jun
100 µm
G
IFE
HF-SCs
DKO*-mT/mG
DKO*K15-mT/mG
Apoptosis
Proliferation
No Apoptosis
Psoriasis-like progression
Time 3-5D
7-10D
15-30D

Figure EV3.

Figure EV4. Isolation of bulge HF-SCs and basal keratinocytes from Co-mT/mG and DKO*-mT/mG ear skin for RNA-seq and gene expression profiling (related to Fig 5).

A Hair follicle stem cells (HF-SC, $CD34^+$ $CD49f^{high}$) and basal keratinocytes (b-KC, $CD49f^{high}$) from ear skin of DKO*-mT/mG collected at day 7 after tamoxifen induction were sorted by FACS for GFP^+ vs. $Tomato^+$. HF-SCs ($CD34^+$ $CD49f^{high}$) and b-KC ($CD49f^{high}$) from control mice were also sorted to sequence the RNA. $n = 3$ mice per condition. Differentially expressed genes were obtained by the comparison of mutant cell populations with their same cell populations from control mice.

B, C Overlapping of differentially expressed genes (DEGs) from the four subpopulations ($HF\text{-}SC^{GFP}$; $HF\text{-}SC^{Tom}$; $b\text{-}KC^{GFP}$; and $b\text{-}KC^{Tom}$) by Venn diagram.

D, E Heat map of up (C)- and down (D)-regulated biological processes based on Gene Ontology IDs for DEGs commonly up- and down-regulated in the four subpopulations ($HF\text{-}SC^{GFP}$; $HF\text{-}SC^{Tom}$; $b\text{-}KC^{GFP}$; and $b\text{-}KC^{Tom}$)

F–L Gene expression for Ptgs2, TNFa, IL-23a, IL-1α, IL-6, IL-1β, and S100a9 in the different epidermal cell subpopulations sorted at mid-term of psoriasis-like development from DKO* mice. $n = 3$ mice. Data represent mean ± SD. Statistical significance $*P < 0.05$, $**P < 0.01$ (Student's two-tailed *t*-test relative to control groups). See Appendix Table S2 for exact *P*-values.

© 2019 The Authors

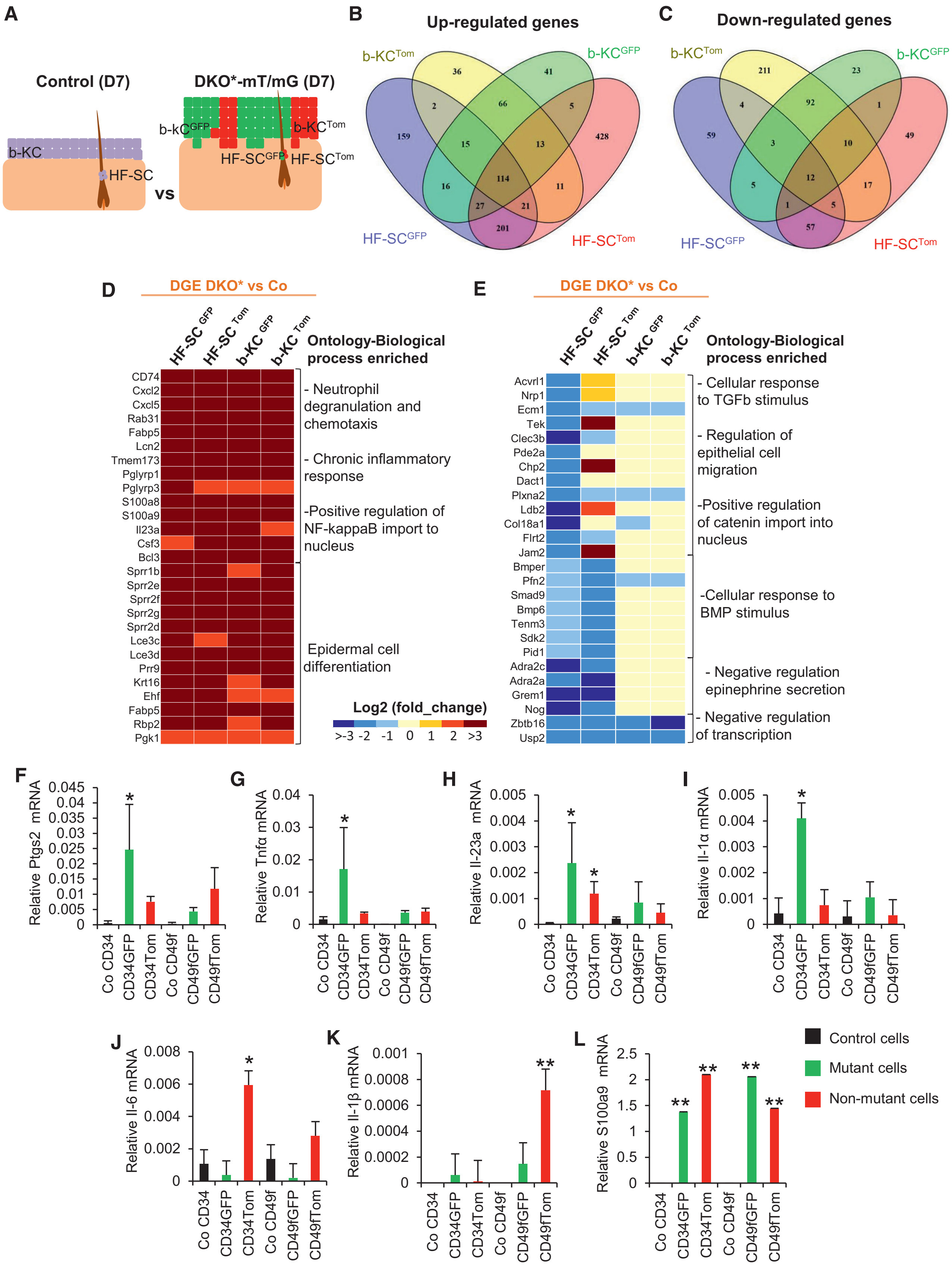
A
Control (D7)
DKO*-mT/mG (D7)
b-KC
HF-SC
vs
b-KC GFP
b-KC Tom
HF-SC GFP
HF-SC Tom
B
Up-regulated genes
C
Down-regulated genes
D
DGE DKO* vs Co
Ontology-Biological process enriched
- Neutrophil degranulation and chemotaxis
- Chronic inflammatory response
-Positive regulation of NF-kappaB import to nucleus
Epidermal cell differentiation
Log2 (fold_change)
>-3 -2 -1 0 1 2 >3
E
DGE DKO* vs Co
Ontology-Biological process enriched
- Cellular response to TGFb stimulus
- Regulation of epithelial cell migration
-Positive regulation of catenin import into nucleus
-Cellular response to BMP stimulus
- Negative regulation epinephrine secretion
- Negative regulation of transcription
F
Relative Ptgs2 mRNA
G
Relative Tnfα mRNA
H
Relative Il-23a mRNA
I
Relative Il-1α mRNA
J
Relative Il-6 mRNA
K
Relative Il-1β mRNA
L
Relative S100a9 mRNA
Co CD34
CD34GFP
CD34Tom
Co CD49f
CD49fGFP
CD49fTom
Control cells
Mutant cells
Non-mutant cells

Figure EV4.

Figure EV5. Regulation of keratinocyte proliferation and pro-inflammatory mediators by TSLP (related to Fig 6).

A Immunofluorescence images of TSLPR (green) and EdU (red) staining in primary keratinocyte cultures from wild-type (WT) mouse skin treated with recombinant TSLP during 48 h.

B Percentage of EdU^+ WT keratinocytes after blocking with recombinant TSLP at different concentrations in culture. WT keratinocytes responded to recombinant TSLP treatment and increased their proliferation. $n = 3$ independent experiments. Data represent mean ± SD. Statistical significance $^{**}P < 0.01$ (two-way ANOVA and Bonferroni post-test). See Appendix Table S2 for exact *P*-values.

C Percentage of EdU^+ WT keratinocytes derived from bulge HF-SCs ($CD34^+$) and basal keratinocytes ($CD49f^+$) after treating with TSLP recombinant in culture. Bulge HF-SCs increased the proliferation rate rather than basal keratinocytes. $n = 2$ independent experiments. Data represent mean ± SD. Statistical significance $^{*}P < 0.05$, $^{***}P < 0.001$ (two-way ANOVA and Bonferroni post-test). See Appendix Table S2 for exact *P*-values.

D Experimental design for induction of $mutant^{GFP}$ KCs *in vitro* and neutralization of secreted TSLP by anti-TSLP. Primary keratinocytes were cultured from the skin of $Junb^{lox/lox}$; $c\text{-}Jun^{lox/lox}$; $Rosa^{Ki/+}$; and $K5cre\text{-}ERT^{+/+}$ mice. 50% of $mutant^{GFP}$ KCs were induced by infection with Cre Adenovirus, and the non-infected cells were non-$mutant^{Tom}$ KCs. Adeno-empty was used as control. After neutralization, GFP^+ and $Tomato^+$ KCs were sorted by FACS and RNA isolation was carried out for further gene expression profiling.

E–M Gene expression profiling of Tslpr, IL-7ra, VEGFα, IL-6, IL-1β, p65, IFN-γ, G-CSF, and S100a9 in sorted GFP vs. Tom KCs after TSLP neutralization. $n = 2$ independent experiments. Data represent mean ± SD. Statistical significance $^{*}P < 0.05$ (Student's two-tailed *t*-test relative to control group). See Appendix Table S2 for exact *P*-value.

 © 2019 The Authors

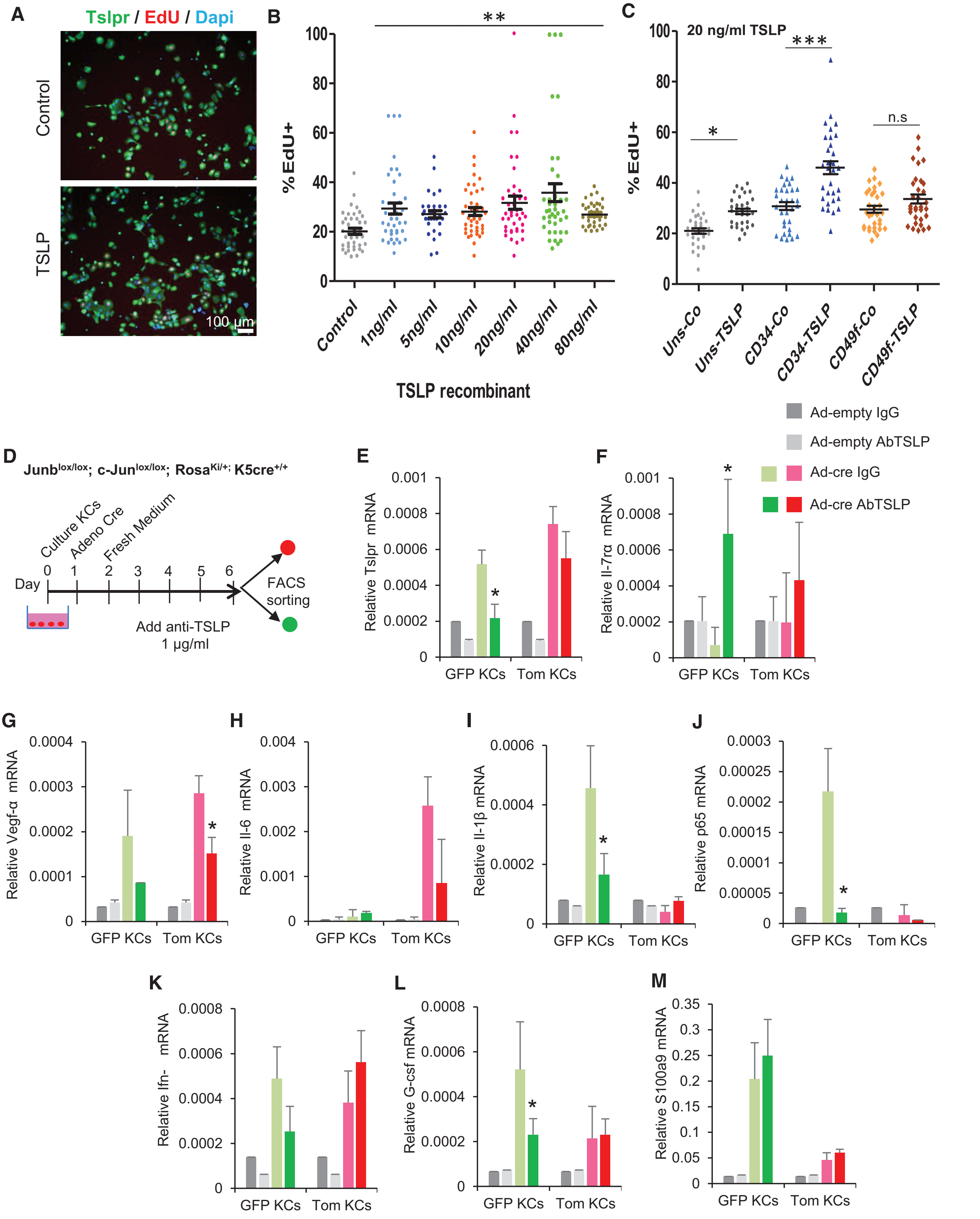
A
Tslpr / EdU / Dapi
Control
TSLP
100 µm
B
% EdU+
Control
1ng/ml
5ng/ml
10ng/ml
20ng/ml
40ng/ml
80ng/ml
TSLP recombinant
C
20 ng/ml TSLP
% EdU+
n.s
Uns-Co
Uns-TSLP
CD34-Co
CD34-TSLP
CD49f-Co
CD49f-TSLP
Ad-empty IgG
Ad-empty AbTSLP
Ad-cre IgG
Ad-cre AbTSLP
D
Junblox/lox; c-Junlox/lox; RosaKi/+; K5cre+/+
Culture KCs
Adeno Cre
Fresh Medium
Day
FACS sorting
Add anti-TSLP 1 µg/ml
E
Relative Tslpr mRNA
F
Relative Il-7rα mRNA
G
Relative Vegf-α mRNA
H
Relative Il-6 mRNA
I
Relative Il-1β mRNA
J
Relative p65 mRNA
K
Relative Ifn- mRNA
L
Relative G-csf mRNA
M
Relative S100a9 mRNA
GFP KCs
Tom KCs

Figure EV5.
