# Supplementary material for: Role of bulge epidermal stem cells and TSLP signaling in psoriasis
Source: EMBO Mol Med. 2019 Sep 26;11(11):e10697. doi: 10.15252/emmm.201910697 (PMC6835205; doi:10.15252/emmm.201910697)
Supplement: Supplementary file 3 — Movie EV1 [file EMMM-11-e10697-s003.zip › Gago-Lopez_et_al-_Legend_Movie_1.docx]

**Role of bulge epidermal stem cells and TSLP signaling in psoriasis**

**Nuria Gago-Lopez et al.**

**Movie EV1. Intravital imaging of ear skin from DKO*-mT/mG mice.** Psoriasis-like DKO*-mT/mG mice at day 5, 7 and 15 after tamoxifen induction were anaesthetized with vaporized isoflurane and placed in a hand-made platform for the mounting of the ear into a coverslip until it was completely flattened and immobile. Mice were provided with vaporized isofluorane at 1.5% through a nose cone for the course of the live imaging session during 8 hours. Elimination of GFP+ epidermal cells were observed at day 7 in the course of 8 hours. At day 15, GFP^+^ epidermal cells were localized around hair follicles.
