# Supplementary material for: Role of bulge epidermal stem cells and TSLP signaling in psoriasis
Source: EMBO Mol Med. 2019 Sep 26;11(11):e10697. doi: 10.15252/emmm.201910697 (PMC6835205; doi:10.15252/emmm.201910697)
Supplement: Supplementary file 4 — Movie EV2 [file EMMM-11-e10697-s004.zip › Gago-Lopez_et_al-_Legend_Movie_2.docx]

**Role of bulge epidermal stem cells and TSLP signaling in psoriasis**

**Nuria Gago-Lopez et al.**

**Movie EV2. Time lapse imaging of primary keratinocyte cultures from ear skin of DKO*-mT/mG mice.**

Representative time lapse movies of keratinocyte colonies derived from purified mutant^GFP^ bulge HF-SCs or b-KCs during 48 hours. Mutant^GFP^ bulge HF-SCs derived keratinocytes grow similarly to control^GFP^ KCs, while mutant^GFP^ b-KCs died.

.
