# Supplementary material for: Role of bulge epidermal stem cells and TSLP signaling in psoriasis
Source: EMBO Mol Med. 2019 Sep 26;11(11):e10697. doi: 10.15252/emmm.201910697 (PMC6835205; doi:10.15252/emmm.201910697)
Supplement: Supplementary file 5 — Movie EV3 [file EMMM-11-e10697-s005.zip › Gago-Lopez_et_al-_Legend_Movie_3.docx]

**Role of bulge epidermal stem cells and TSLP signaling in psoriasis**

**Nuria Gago-Lopez et al.**

**Movie EV3. Time lapse imaging of primary keratinocyte co-cultures from ear skin of DKO*-mT/mG mice.**

Representative time lapse movies of mutant^GFP^ and non-mutant^Tom^ keratinocyte co-cultures derived from bulge HF-SCs or b-KCs during 48 hours. Non-mutant^Tom^ KCs derived from bulge HF-SCs or b-KCs increased the proliferation in contact with mutant^GFP^ KCs.
